# Carotenoids in Algae: Distributions, Biosyntheses and Functions

**DOI:** 10.3390/md9061101

**Published:** 2011-06-15

**Authors:** Shinichi Takaichi

**Affiliations:** Department of Biology, Nippon Medical School, Kosugi-cho, Nakahara, Kawasaki 211-0063, Japan; E-Mail: takaichi@nms.ac.jp; Tel.: +81-44-733-3584; Fax: +81-44-733-3584

**Keywords:** algal phylogeny, biosynthesis of carotenoids, distribution of carotenoids, function of carotenoids, pigment-protein complex

## Abstract

For photosynthesis, phototrophic organisms necessarily synthesize not only chlorophylls but also carotenoids. Many kinds of carotenoids are found in algae and, recently, taxonomic studies of algae have been developed. In this review, the relationship between the distribution of carotenoids and the phylogeny of oxygenic phototrophs in sea and fresh water, including cyanobacteria, red algae, brown algae and green algae, is summarized. These phototrophs contain division- or class-specific carotenoids, such as fucoxanthin, peridinin and siphonaxanthin. The distribution of α-carotene and its derivatives, such as lutein, loroxanthin and siphonaxanthin, are limited to divisions of Rhodophyta (macrophytic type), Cryptophyta, Euglenophyta, Chlorarachniophyta and Chlorophyta. In addition, carotenogenesis pathways are discussed based on the chemical structures of carotenoids and known characteristics of carotenogenesis enzymes in other organisms; genes and enzymes for carotenogenesis in algae are not yet known. Most carotenoids bind to membrane-bound pigment-protein complexes, such as reaction center, light-harvesting and cytochrome *b*_6_*f* complexes. Water-soluble peridinin-chlorophyll *a*-protein (PCP) and orange carotenoid protein (OCP) are also established. Some functions of carotenoids in photosynthesis are also briefly summarized.

## 1. Introduction

Algae are classified throughout many divisions of the Kingdom Plantae. Their sizes range from single cells of picophytoplankton—the smallest of which are less than 1 μm—to seaweeds, the largest of which are more than 50 m. Attempts have been made to cultivate single-cell algae for a long time, but numbers were limited. With the recent development of culture techniques, some single-cell species can be cultured, and their characteristics, including pigments, can be studied. With the development of taxonomic technology, including DNA base sequences of 16S or 18S rRNA and some genes, algae phylogenetics has been developed.

More than 750 structurally defined carotenoids are reported from nature; land plants, algae, bacteria including cyanobacteria and photosynthetic bacteria, archaea, fungus and animals [1]. Except for animals, these organisms can synthesize many kinds of carotenoids, which are synthesized from diverse carotenogenesis pathways. These carotenoids and carotenogenesis pathways can be used as chemotaxonomic markers [2–7]. In addition, characteristics of carotenogenesis enzymes and genes are investigated. Some carotenogenesis genes have high similarity from bacteria to land plants, but some have low similarity. Some homologous genes have been proposed [8,9], but some carotenogenesis enzymes and genes, especially algae-specific ones, are not found.

In this review, the term algae refers to an oxygenic phototroph found in both seawater and fresh water, including cyanobacteria but excluding land plants. Distribution of carotenoids, carotenogenesis enzymes and pathways, and function of carotenoids in photosynthesis in algae are summarized.

## 2. Distribution of Carotenoids

**Figure 1:**
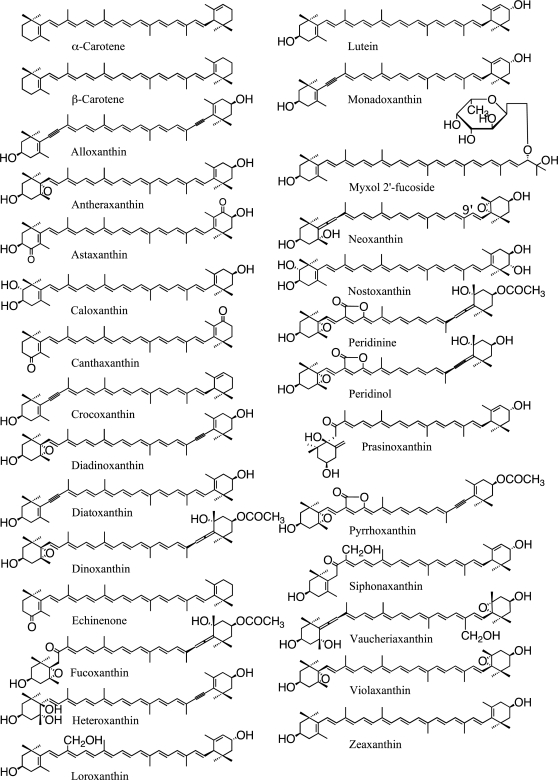
Structures of some carotenoids.

Many different kinds of carotenoids were found from the algal species studied. Structures of some important carotenoids in algae are illustrated in Figure 1. Among them, approximately 30 types may have functions in photosynthesis, and others may be intermediates of carotenogenesis or accumulated carotenoids. Some carotenoids are found only in some algal divisions or classes; therefore, these carotenoids and also chlorophylls can be used as chemotaxonomic markers, and their distribution in algae is summarized in Table 1 [2–6].

Allene (C═C═C) is a unique structure in natural products, and is found mainly in carotenoids [10]; fucoxanthin in brown algae and diatoms, 19′-acyloxyfucoxanthin in Haptophyta and Dinophyta, peridinin only in dinoflagellates, and 9′-*cis* neoxanthin in green algae and land plants. Acetylene (C≡C) is also a unique structure, and acetylenic carotenoids are found only in algae; alloxanthin, crocoxanthin and monadoxanthin in Cryptophyta, and diadinoxanthin and diatoxanthin in Heterokontophyta, Haptophyta, Dinophyta and Euglenophyta. Acetylated carotenoids (-O-CO-CH_3_), such as fucoxanthin, peridinin and dinoxanthin, are also mainly found in algae, such as Heterokontophyta, Haptophyta and Dinophyta. These carotenoids are specific to certain algal divisions and classes, and they are summarized in Table 1 based on our results [11–14] and some references [1–6].

Many cyanobacteria contain β-carotene, zeaxanthin, echinenone and myxol pentosides (myxoxanthophyll), while some species lack part of these and some contain additional carotenoids, such as nostoxanthin, canthaxanthin and oscillol dipentoside (Table 1, Figure 1) [13]. In addition, the carotenoid compositions of cyanobacteria are very different from those of chloroplasts in algae; consequently, during symbiosis of cyanobacteria to eukaryotic cells, carotenoids might be considerably restructured [13]. Note that since the name of myxoxanthophyll cannot specify the glycoside moieties, we have proposed the name of myxol glycosides to specify the glycosides, such as myxol 2′-α-l-fucoside, 4-ketomyxol 2′-rhamnoside and oscillol dichinovoside [13,15].

Rhodophyta (red algae) can be divided into two groups based on carotenoid composition; the unicellular type contains only β-carotene and zeaxanthin, and the macrophytic type contains additional α-carotene and lutein (Table 1, Figure 1) [16]. The relationship between phylogenetics of red algae and carotenoid composition is not clear [14]. Cryptophyta also contains α-carotene and its acetylenic derivatives, crocoxanthin and monadoxanthin, which are only found in this division.

Heterokontophyta, Haptophyta and Dinophyta contain β-carotene and its derivatives as well as chlorophyll *c* (Table 1, Figure 1). These divisions, except for Eustigmatophyceae, which lacks chlorophylls *c*, contain unique acetylenic carotenoids of diadinoxanthin and diatoxanthin. Fucoxanthin and its derivatives are found in only four classes of Heterokontophyta (Chrysophyceae, Raphidophyceae, Bacillariophyceae and Phaeophyceae), Haptophyta and Dinophyta. Peridinin and its derivatives are found only in Dinophyta. Fucoxanthin and peridinin have unique structures (Figure 1) and are class-specific carotenoids (Table 1).

Euglenophyta, Chlorarachniophyta and Chlorophyta contain the same carotenoids, such as β-carotene, violaxanthin, 9′-*cis* neoxanthin [11] and lutein, as well as chlorophyll *a* and *b* with land plants (Table 1, Figure 1). Some classes contain additional carotenoids, such as loroxanthin, siphonaxanthin and prasinoxanthin, which are derivatives of lutein, and are class specific.

Note that identifications of some carotenoids were lacking because of insufficient analysis, and that some algae names were changed because of new developments in taxonomic technology and phylogenetic classification.

## 3. Carotenogenesis Pathways, Enzymes and Genes

**Figure 2:**
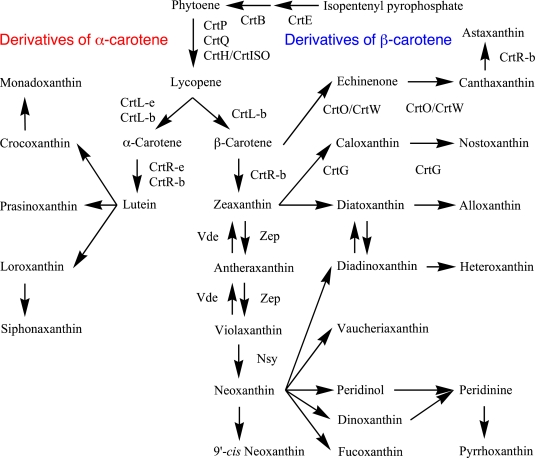
Carotenogenesis pathways and enzymes, whose functions are confirmed, in oxygenic phototrophs.

**Figure 3:**
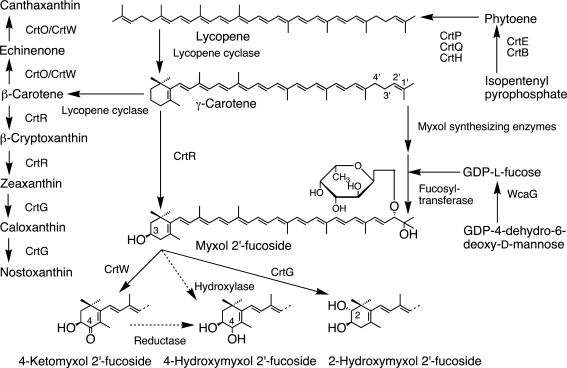
Carotenogenesis pathways and enzymes in cyanobacteria.

Carotenogenesis pathways and their enzymes are mainly investigated in cyanobacteria [13] and land plants among oxygenic phototrophs [17]. Especially in land plants, carotenogenesis pathways and characteristics of enzymes are studied in detail (Figure 2). On the other hand, algae have common pathways with land plants and also additional algae-specific pathways, which are solely proposed based on the chemical structures of carotenoids (Figure 2). Some common carotenogenesis genes in algae are suggested from homology of the known genes [8,9], but most genes and enzymes for algae-specific pathways are still unknown (Figure 2). In cyanobacteria, since carotenoid compositions are different from those in land plants and algae, the pathways and enzymes are also different from those in Figure 2, and they are shown in Figure 3. In addition, carotenogenesis enzymes and genes, whose functions are confirmed in algae, including cyanobacteria, are summarized in Table 2. Unfortunately, these enzymes are mostly from cyanobacteria and green algae (Table 2).

### 3.1. Lycopene Synthesis

#### 3.1.1. Isopentenyl Pyrophosphate to Phytoene Synthesis

Isopentenyl pyrophosphate (IPP), a C_5_-compound, is the source of isoprenoids, terpenes, quinones, sterols, phytol of chlorophylls, and carotenoids. There are two known independent pathways of IPP synthesis: the classical mevalonate (MVA) pathway and the alternative, non-mevalonate, 1-deoxy-d-xylulose-5-phosphate (DOXP) pathway [18,19]. In the MVA pathway, acetyl-Coenzyme A is converted to IPP through mevalonate, and the enzymes and genes are well studied [20]. The pathway is found in plant cytoplasm, animals and some bacteria [18,20]. The DOXP pathway was found in the 1990s, and in this pathway, pyruvate and glycelaldehyde are converted to IPP. The DOXP pathway is found in cyanobacteria, the plastids of algae and land plants, and some bacteria [18]. Carotenoids are synthesized in plastids. Exceptionally among oxygenic phototrophs, Euglenophyceae has only the MVA pathway, and Chlorophyceae has only the DOXP pathway [18].

Most carotenoids consist of eight IPP units. Farnesyl pyrophosphate (C_15_) is synthesized from three IPPs, after which one IPP is added to farnesyl pyrophosphate by geranylgeranyl pyrophosphate synthase (CrtE, GGPS) to yield geranylgeranyl pyrophosphate (C_20_). In a head-to-head condensation of the two C_20_ compounds, the first carotene, phytoene (C_40_), is formed by phytoene synthase (CrtB, Pys, Psy) using ATP [57,58]. This pathway has been confirmed by cloning genes from two species of *Rhodobacter* (purple bacteria) and two species of *Pantoea* (previously *Erwinia*) [57–59]. Among oxygenic phototrophs, the functions of CrtE of *Thermosynechococcus elongatus* BP-1 [21], and CrtB of three species of cyanobacteria [22–24] and two species of green algae [25,26] have also been confirmed (Table 2). The *crtE* and *crtB* genes have high sequence similarity from bacteria to land plants, respectively.

#### 3.1.2. Phytoene to Lycopene Synthesis

Four desaturation steps are needed in the conversion from phytoene to lycopene. Oxygenic phototrophs require three enzymes: phytoene desaturase (CrtP, Pds), ζ-carotene desaturase (CrtQ, Zds) and *cis*-carotene isomerase (CrtH, CrtISO) (Figure 2). CrtP catalyzes the first two desaturation steps, from phytoene to ζ-carotene through phytofluene, and CrtQ catalyzes two additional desaturation steps, from ζ-carotene to lycopene through neurosporene. During desaturation by CrtQ, neurosporene and lycopene are isomerized to poly-*cis* forms, and then CrtH isomerizes to all-*trans* forms. Light is also effective for their photoisomerization to all-*trans* forms [34]. The functions of these enzymes have been mainly confirmed in cyanobacteria, green algae and land plants (Table 2): CrtP from *Synechocystis* sp. PCC 6803 [28], *Synechococcus elongatus* PCC 7942 [23], *Chlamydomonas reinhardtii* [29] and *Chlorella zofingiensis* [30,31], CrtQ from *Anabaena* sp. PCC 7120 (CrtQa, *crtI*-like sequence) [32] and *Synechocystis* sp. PCC 6803 (CrtQb, plant *crtQ*-like) [33], and CrtH from *Synechocystis* sp. PCC 6803 [34,35]. The CrtP of *S. elongatus* PCC 7942 is stimulated by NAD(P) and oxygen as a possible final electron acceptor [60]. CrtQa has sequence homology with bacterial phytoene desaturase (CrtI) and CrtH, while CrtQb has sequence homology with CrtP. In addition, genes homologous to *crtQa* are not found in cyanobacteria; therefore, among oxygenic phototrophs, *Anabaena* sp. PCC 7120 is the only species to have functional CrtQa.

In contrast, the bacterial type uses only one enzyme, phytoene desaturase (CrtI), to convert from phytoene to lycopene, and the primitive cyanobacterium of *Gloeobacter violaceus* PCC 7421 uses this type of CrtI, and the homologous genes of *crtP*, *crtQ* and *crtH* are not found in the genome [22,27]; therefore, *G. violaceus* is the first oxygenic phototroph that has been shown to use this type (Table 2). These observations suggest the following evolutionary scheme for this step in the reaction: the desaturation of phytoene was initially carried out by CrtI in ancestral cyanobacteria, *crtP* and related desaturase genes were acquired, and ultimately, there was replacement of *crtI* by *crtP* [27]. Among anoxygenic phototrophs, purple bacteria, green filamentous bacteria and heliobacteria use CrtI, whereas green sulfur bacteria use CrtP, CrtQ and CrtH [61].

### 3.2. β-Carotene and α-Carotene Synthesis by Lycopene Cyclases

All carotenoids in oxygenic phototrophs are dicyclic carotenoids; β-carotene, α-carotene and their derivatives, are derived from lycopene (Figures 1 and 2). Exceptionally, myxol glycosides and oscillol diglycosides in cyanobacteria are monocyclic and acyclic carotenoids, respectively.

Lycopene is cyclized into either β-carotene through γ-carotene, or α-carotene through γ-carotene or δ-carotene. Three distinct families of lycopene cyclases have been identified in carotenogenetic organisms [13,62,63]. One large family contains CrtY in some bacteria except cyanobacteria, and CrtL (CrtL-b, Lcy-b) in some cyanobacteria and land plants. Lycopene ɛ-cyclases (CrtL-e, Lcy-e) from land plants and lycopene β-monocyclases (CrtYm, CrtLm) from bacteria are also included. Their amino acid sequences exhibit a significant five conserved regions [39,62,64], and have an NAD(P)/FAD-binding motif [65]. Note that Maresca *et al.* [63] divide this family into two CrtY and CrtL families. Three enzymes from Rhodophyta, *Cyanidioschyzon merolae* [38], and Chlorophyceae, *Dunaliella salina* [39] and *Haematococcus pluvialis* [40], are functionally confirmed (Table 2).

Some cyanobacteria also contain these enzymes (Table 2). *Synechococcus elongatus* PCC 7942 contains a functional CrtL [36]. *Prochlorococcus marinus* MED4 contains two lycopene cyclases (Table 2), which have sequence homology to CrtL. CrtL-b exhibits lycopene β-cyclase activity, while CrtL-e is a bifunctional enzyme having both lycopene ɛ-cyclase and lycopene β-cyclase activities [37]. The combination of these two cyclases allows the production of β-carotene, α-carotene and ɛ-carotene. Both enzymes might have originated from the duplication of a single gene. The characteristics of this CrtL-e are somewhat different from those in land plants [66]. In addition, the β-end groups of both β-carotene and α-carotene (left half) might be hydroxylated by CrtR to zeaxanthin through β-cryptoxanthin and 3-hydroxy-α-carotene, respectively, in *P. marinus. Acaryochloris marina* MBIC 11017, which produces α-carotene, contains only one *crtL*-like gene from genome sequence [14].

The second family of lycopene cyclases, heterodimer (*crtYc* and *crtYd*) or monomer (*crtYc-Yd*), has been found in some bacteria, archaea and fungi [62,67], but not in phototrophs.

Recently, a new family of functional lycopene cyclase, CruA, has been found in *Chlorobaculum* (previously *Chlorobium*) *tepidum* (green sulfur bacterium), and the main product is γ-carotene in *Escherichia coli*, which produces lycopene [68]. Homologous genes, *cruA* and *cruP*, have been found in the genome of *Synechococcus* sp. PCC 7002, and their main products are γ-carotene, in *E. coli*, which produces lycopene [63]. In addition, their homologous genes are widely distributed in cyanobacteria, such as *Synechocystis* sp. PCC 6803 and *Anabaena* sp. PCC 7120; however, these *cruA*- and *cruP*-like genes from both *Synechocystis* sp. PCC 6803 and *Anabaena* sp. PCC 7120 did not show the lycopene dicyclase or monocyclase activities [14]. *S. elongatus* PCC 6301 and PCC 7942, and *A. marina* MBIC 11017 contain *crtL*-, *cruA*- and *cruP*-like genes; consequently, distributions of functional lycopene cyclases (CrtL-, CruA- and CruP-like) in cyanobacteria are unknown.

Since *Synechocystis* sp. PCC 6803 and *Anabaena* sp. PCC 7120 lack *crtL*-like genes and contain non-functional *cruA*-like genes, there is a possibility to present a fourth new family of lycopene cyclases in these cyanobacteria. Further studies of distributions of functional lycopene cyclases (CrtL- and CruA-like, or others) in cyanobacteria are needed.

Distribution of α-carotene, that is, CrtL-e, is limited in some algae classes (Table 1). Genes and enzymes of CrtL-e are not found in algae. In some species of land plants, the characteristics of CrtL-e were investigated [66], and were shown to have sequence homology with *crtL-b*. Lycopene is first converted to ζ-carotene by CrtL-e, and then to α-carotene by CrtL-b. γ-Carotene produced by CrtL-b is not a suitable substrate for CrtL-e.

### 3.3. β-Carotene Derivatives and Their Synthesis

#### 3.3.1. Cyanobacteria

Some cyanobacteria produce zeaxanthin, and some produce both zeaxanthin and nostoxanthin (Figure 3). First, the C-3 and C-3′ hydroxyl groups of zeaxanthin are introduced to β-carotene by β-carotene hydroxylase (CrtR) through β-cryptoxanthin. Then, the C-2 and C-2′ hydroxyl groups of nostoxanthin are introduced by 2,2′-β-hydroxylase (CrtG) through caloxanthin (Table 2) [13,41–43,47]. The same enzymes, CrtR and CrtG, can also introduce hydroxyl groups to deoxymyxol and myxol to produce myxol and 2-hydroxymyxol, respectively [13,44,47]; consequently, the same enzymes are used in two pathways.

Cyanobacteria contain two ketocarotenoids, namely, canthaxanthin and 4-ketomyxol. Two distinct β-carotene ketolases, CrtO and CrtW, are known, and only seven enzymes are functionally confirmed in four species of cyanobacteria (Table 2) [13]. CrtO catalyzes β-carotene to echinenone, and the final product is canthaxanthin [22,42,45,50,51]. CrtW can introduce a keto group into β-carotene, zeaxanthin and myxol to produce canthaxanthin, astaxanthin and 4-ketomyxol, respectively (Figure 3) [22,27,42,50,52]; therefore, these ketolases are properly used in two pathways, β-carotene and myxol, depending on the species [13].

The pathway and the enzymes to produce the right half of myxol 2′-pentoside are still unknown (Figure 3) [13].

#### 3.3.2. Land Plants

In land plants, most of the carotenogenesis pathways and the functionally confirmed enzymes are known (Figure 2). Hydroxyl groups are introduced into β-carotene to produce zeaxanthin by β-carotene hydroxylase (CrtR, CrtR-b, BCH). Epoxy groups are introduced into zeaxanthin by zeaxanthin epoxidase (Zep, NPQ) to produce violaxanthin through antheraxanthin. Under high light conditions, violaxanthin is changed into zeaxanthin by violaxanthin de-epoxidase (Vde) for dispersion of excess energy from excited chlorophylls. One end group of violaxanthin is changed to an allene group of neoxanthin by neoxanthin synthase (Nsy). Because all neoxanthin in chloroplasts has the 9′-*cis* form, unknown 9′-isomerase for all *trans* neoxanthin to 9′-*cis* neoxanthin should be present [11].

#### 3.3.3. Algae

Little is known for the carotenogenesis pathways among algae, but some are proposed based on the chemical structures of carotenoids (Figure 2). Functionally confirmed enzymes are mainly reported in Chlorophyceae including *Chlorella*, *Chlamydomonas*, *Dunaliella* and *Haematococcus* for CrtB, CrtP, CrtL-b, CrtR-b [46], Zep [48], Vde [49], and CrtW (Table 2).

In the cell-free preparation of *Amphidinium carterae* (Dinophyta), ^14^C-labellled zeaxanthin was incorporated into allenic carotenoid of neoxanthin, and then into acetylenic diadinoxanthin and C_37_ peridinin (Figure 2). In addition, the three carbon atoms of C-13′,14′,20′ of peridinin were eliminated from neoxanthin (C-13,14,20) [69,70]. In organic chemistry, the C-7,8 double bond of zeaxanthin can be oxidized to the triple bond (acetylene group) of diatoxanthin [17].

Allenic carotenoids are very limited in algae. From their chemical structures, all *trans* neoxanthin might be changed to fucoxanthin, dinoxanthin, peridinin, vaucheriaxanthin and diadinoxanthin, but the pathways and enzymes are still unknown (Figures 1 and 2).

Under a stressful environment, such as high light, UV irradiation and nutrition stress, some Chlorophyceae, such as *Haematococcus*, *Chlorella* and *Scenedesmus*, accumulate ketocarotenoids, canthaxanthin and astaxanthin, which are synthesized by combining CrtR-b and β-carotene ketolase (CrtW, BKT) (Table 2) [53–56,71]. Note that although β-carotene ketolase of *Haematococcus* and *Chlorella* were named CrtO at first [53,56], they are CrtW-type not CrtO-type from amino acid sequences (Table 2).

### 3.4. α-Carotene Derivatives and Their Synthesis

In *Arabidopsis thaliana*, β-carotene is hydroxylated mainly by the non-heme di-iron enzymes, BCH1 and BCH2 (CrtR-b), to produce zeaxanthin, while α-carotene is mainly hydroxylated by the cytochrome P450 enzymes, CYP97A3 for the β-end group and CYP97C1 for the β- and ɛ-end groups, to produce lutein [72].

Lutein and its derivatives are found only in Rhodophyta (macrophytic type), Cryptophyta, Euglenophyta, Chlorarachniophyta and Chlorophyta (Table 1), but nothing is known for hydroxylation of α-carotene. From the chemical structures of siphonaxanthin [12], loroxanthin, prasinoxanthin and monadoxanthin, it could be considered that they are derived from lutein, but the pathways and enzymes are still unknown (Figures 1 and 2).

## 4. Function of Carotenoids

For photosynthesis, both carotenoids and chlorophylls are necessarily bound to peptides to form pigment-protein complexes in the thylakoid membrane. Five main kinds of the complexes described below are isolated from some algae, and the pigment compositions are investigated [73–75]. Exceptionally in cyanobacteria, myxol glycosides and some carotenoids are located in the cytoplasmic membrane for protection from high-light [76,77].

β-Carotene is presented in the most divisions of the reaction-center complexes (RC) and the light-harvesting complexes (LHC) of photosystem I (PSI) as well as the RC and the core LHC of photosystem II (PSII); exceptionally zeaxanthin is presented in some red algae of the LHC of PSI. On the other hand, in the peripheral LHC of PSII, the bound carotenoids are heterogenous depending on the classes. Major carotenoids are alloxanthin (Cryptophyta); fucoxanthin (Chrysophyceae, Raphidophyceae, Bacillariophyceae, Phaeophyceae and Haptophyta); diadinoxanthin and vaucheriaxanthin (Xanthophyceae); violaxanthin and vaucheriaxanthin (Eustigmatophyceae); peridinin (Dinophyta); diadinoxanthin (Euglenophyta); siphonaxanthin (Chlorophyceae and Ulvophyceae); and lutein, violaxanthin and 9′-*cis* neoxanthin (land plants) (Figure 1) [73–75]. β-Carotene in both RC might have protective functions, and carotenoids in the peripheral LHC of PSII mainly might have light-harvesting functions.

The dimeric cytochrome *b*_6_*f* complexes of the cyanobacterium *Mastigocladus laminosus* [78] and the green alga *Chlamydomonas reinhardtii* [79] contain two β-carotene and two chlorophyll *a* molecules, while that of the cyanobacterium *Synechocystis* sp. PCC 6803 contains two echinenone and two chlorophyll *a* molecules [80]. These carotenoids might have protective functions.

The water-soluble peripheral LHC of peridinin-chlorophyll-protein (PCP) isolated from *Amphidinium carterae* (Dinophyta) has a trimeric structure, and the monomer contains eight peridinin and two chlorophyll *a* molecules [81]. The water-soluble orange carotenoid protein (OCP) isolated from the cyanobacterium *Arthrospira maima* forms a homodimer with two 3′-hydroxyechinenone molecules [82]. OCP is also found in some cyanobacteria, and its function might regulate energy dissipation from phycobilisomes to PSII [83].

The keto groups at C-8 of fucoxanthin [84], siphonaxanthin [85,86] and prasinoxanthin [87], which are found only in algae, are the single-bond *trans*-conformation for the conjugated double bond (Figure 1). From the femtosecond time-resolved fluorescence spectroscopy of the purified carotenoids in organic solvents and the LHC in solution, these keto-carotenoids and peridinin have been found to have highly efficient energy transfer from the S_1_ state, not the S_2_ state, of carotenoids to chlorophylls. From the comparison of other structural carotenoids, these keto groups are essential for high efficiency [88,89]. These keto-carotenoids mainly might have light-harvesting functions.

The xanthophyll cycle, also known as the violaxanthin cycle, is the cyclical interconversion of violaxanthin, antheraxanthin and zeaxanthin in green algae and land plants (Figure 2) [90]. Zep catalyzes zeaxanthin to violaxanthin through antheraxanthin during biosynthesis. Violaxanthin is found in the peripheral LHC of PSII. Under high light conditions, Vde is activated and catalyzes de-epoxidation of violaxanthin to zeaxanthin through antheraxanthin. Zeaxanthin is used for the dissipation of excess energy from excited chlorophylls. Zep from Chlorophyceae *Chlamydomonas reinhardtii* [48] and Vde from Pracinophyceae *Mantonilla squamata* [49] are functionally confirmed (Table 2). Similarly, the diadinoxanthin cycle occurs in Heterokontophyta, Haptophyta and Dinophyta, which contain diadinoxanthin and diatoxanthin (Figure 2). The enzymes of diadinoxanthin de-epoxidase and diatoxanthin epoxidase have not yet been found [9,91], but the characteristics of partially purified diadinoxanthin de-epoxidase from the diatom *Cyclotella meneghinaina* are reported [92].

## Figures and Tables

**Table 1:** Distribution of carotenoids in algae.

**Division** **Class**	**Carotene**		**Xanthophyll**											**Chlorophyll**		
		
	**β**	**α**	**Ze**	**Vi**	**Ne**	**Da**	**Dd**	**Fx**	**Va**	**Lu**	**Lo**	**Sx**	**Other xanthophyll(s)**	***a***	***b***	***c***
Cyanophyta	H	L	H										No, L; Ec, H; My, H	H	L	

Glaucophyta	H		H											H		

Rhodophyta																
Unicellular type	H		H											H		
Macrophytic type	L	L	H	L				L		H				H		

Cryptophyta		H	L										Al, L; Cr, L; Mo, L	H		H

Heterokontophyta																
Chrysophyceae	H		L			L	L	H	L					H		H
Raphidophyceae	H		H	L		L	L	L						H		H
Bacillariophyceae	H		L			L	L	H						H		H
Phaeophyceae	H		H	H		L	L	H						H		H
Xanthophyceae	H		L			H	H						Va-FA, L	H		H
Eustigmatophyceae	H			H					L					H		

Haptophyta	H		L			L	H	H					Fx-FA, L	H		H

Dinophyta	L		L			L	H	L					Pe, H	H		H

Euglenophyta	H		L		L	L	H				L	L		H	H	

Chlorarachniophyta	H		L	L	L					L	L		Lo-FA, L	H	H	

Chlorophyta																
Prasinophyceae	H	L	L	H	H					L	L	H	Pr, L; Lo-FA, L; Sx-FA, H	H	H	
Chlorophyceae	H	H	L	H	H					H	L	L	Sx-FA, L	H	H	
Ulvophyceae	H	L	L	H	H					L	L	L	Sx-FA, H	H	H	
Trebouxiophyceae	H		L	H	H					H				H	H	
Charophyceae	H		L	H	H					H				H	H	

Land Plants	H	L	L	H	H					H				H	H	

H, Major carotenoid in most species of the class; L, Low content in most species or major carotenoid in some species. α, α-carotene; β, β-carotene; Al, alloxanthin; Cr, crocoxanthin; Da, diatoxanthin; Dd, diadinoxanthin; Ec, echinenone; -FA, fatty acid ester; Fx, fucoxanthin; Lo, loroxanthin; Lu, lutein; Mo, monadoxanthin; My, myxol glycosides and oscillol glycosides; Ne, neoxanthin; No, nostoxanthin; Pe, peridinin; Pr, prasinoxanthin; Sx, siphonaxanthin; Va, vaucheriaxanthin; Vi, violaxanthin; Ze, zeaxanthin. Red, α-carotene and its derivatives.

**Table 2:** Carotenogenesis genes and enzymes, whose functions are confirmed, in algae.

**Gene**	**Enzyme**	**Species**	**References**
*crtE*, *ggps*	Geranylgeranyl pyrophosphate synthase	*Thermosynechococcus elongates* BP-1	[21]

*crtB*, *pys*, *psy*	Phytoene synthase	*Gloeobacter violaceus* PCC 7421	[22]
		*Synechococcus elongatus* PCC 7942	[23]
		*Synechocystis* sp. PCC 6803	[24]
		*Chlamydomonas reinhardtii*	[25]
		*Haematococcus pluvialis* NIES-144	[26]

*crtI*	Phytoene desaturase (bacterial type)	*Gloeobacter violaceus* PCC 7421	[22,27]

*crtP*, *pds*	Phytoene desaturase (plant type)	*Synechococcus elongatus* PCC 7942	[23]
		*Synechocystis* sp. PCC 6803	[28]
		*Chlamydomonas reinhardtii*	[29]
		*Chlorella zofingiensis* ATCC 30412	[30,31]

*crtQ*, *zds*	ζ-Carotene desaturase	*Anabaena* sp. PCC 7120	[32]
		*Synechocystis* sp. PCC 6803	[33]

*crtH*, *crtISO*	Carotene isomerase	*Synechocystis* sp. PCC 6803	[34,35]

*crtL*, *crtL-b*, *lcy-b*	Lycopene β-cyclase	*Synechococcus elongatus* PCC 7942	[36]
		*Prochlorococcus marinus* MED4	[37]
		*Cyanidioschyzon merolae* NIES-1332	[38]
		*Dunaliella salina* CCAP 19/30	[39]
		*Haematococcus pluvialis* NIES-144	[40]

*crtL-e*, *lcy-e*	Lycopene ɛ-cyclase	*Prochlorococcus marinus* MED4	[37]

*crtR*	β-Carotene hydroxylase	*Anabaena* sp. PCC 7120	[41,42]
		*Anabaena variabilis* ATCC 29413	[42]
		*Synechocystis* sp. PCC 6803	[42–45]
		*Haematococcus pluvialis* NIES-144	[46]

*crtG*	β-Carotene 2-hydroxylase	*Thermosynechococcus elongates* BP-1	[47]

*zep*, *npq*	Zeaxanthin epoxidase	*Chlamydomonas reinhardtii* CC-125	[48]

*vde*	Violaxanthin de-epoxidase	*Mantonilla squamata*	[49]

*crtO*	β-Carotene ketolase	*Anabaena* sp. PCC 7120	[50]
		*Gloeobacter violaceus* PCC 7421	[22]
		*Synechocystis* sp. PCC 6803	[42,45,51]

*crtW*, *bkt*	β-Carotene ketolase	*Anabaena* sp. PCC 7120	[42,50]
		*Gloeobacter violaceus* PCC 7421	[22,27,42]
		*Nostoc punctiforme* PCC 73102	[42,52]
		*Chlorella zofingiensis* ATCC 30412	[53]
		*Haematococcus pluvialis* NIES-144	[54,55]
		*Haematococcus pluvialis* strain 34/7	[56]

Red, genes and enzymes related to α-carotene.
